# Traumatic Posterior Atlanto-occipital Dislocation With Three-part Jefferson Fracture and Subaxial Distractive Extension Injury

**DOI:** 10.5435/JAAOSGlobal-D-21-00070

**Published:** 2021-07-16

**Authors:** Dong-Gune Chang, Jae Won Lee, Jong-Beom Park, Jaehoon Kim

**Affiliations:** From the Department of Orthopaedic Surgery (Dr. Chang), Sanggye Paik Hospital, College of Medicine, Inje University, Seoul, Korea, and the Department of Orthopaedic Surgery (Dr. Lee, Dr. Park, and Dr. Kim), College of Medicine, The Catholic University of Korea, Seoul, Korea.

## Abstract

No previous reports have described combined upper and lower cervical injuries caused by a contrary injury mechanism.

A 44-year-old man was transferred complaining of quadriplegia caused by a rear-end collision car accident. CT and MRI findings revealed posterior atlanto-occipital dislocation (AOD) with three-part Jefferson fracture and subaxial distractive extension (DE) injury at the C3-4 and C6-7 levels. MRI showed spinal cord injury at C3-4 and C6-7, which caused quadriplegia and respiratory failure.

When the patient arrived at the emergency department, he was already intubated because of respiratory failure. The patient was also hemodynamically unstable after lung injury and pelvic bone fracture. The patient died 1 day after the accident before undergoing surgical intervention.

To the best of our knowledge, this is the first report of a case of a traumatic posterior AOD with three-part Jefferson fracture and subaxial DE injury caused by a contrary injury mechanism. Subaxial DE injury, not posterior AOD, caused fatal situation in this case. High index of suspicion and careful radiologic examination are needed to investigate the presence of associated lower cervical spine injury caused by a contrary injury mechanism in traumatic posterior AOD, which may affect treatment, outcome, and prognosis.

Traumatic atlanto-occipital dislocation (AOD) is a rare and usually fatal injury.^1-3^ AOD occurs either alone or in conjunction with other spinal injuries, especially upper cervical spine injuries.^3^ To date, four types of AODs have been reported: anterior, vertical (or distractive), posterior, and lateral.^4^ The injury mechanism of posterior-type AOD is usually axial loading compression, and any associated upper and lower cervical injuries are often caused by the same injury mechanism, such as Jefferson fracture or subaxial compressive flexion injury.^5^

However, no previous reports have described combined upper and lower cervical injury caused by a contrary injury mechanism. Therefore, in this study, we report a very rare case of traumatic AOD with three-part Jefferson fracture and subaxial distractive extension (DE) injury at C3-4 and C6-7 caused by a rear-end collision car accident.

## Case Report

**Figure 1:**
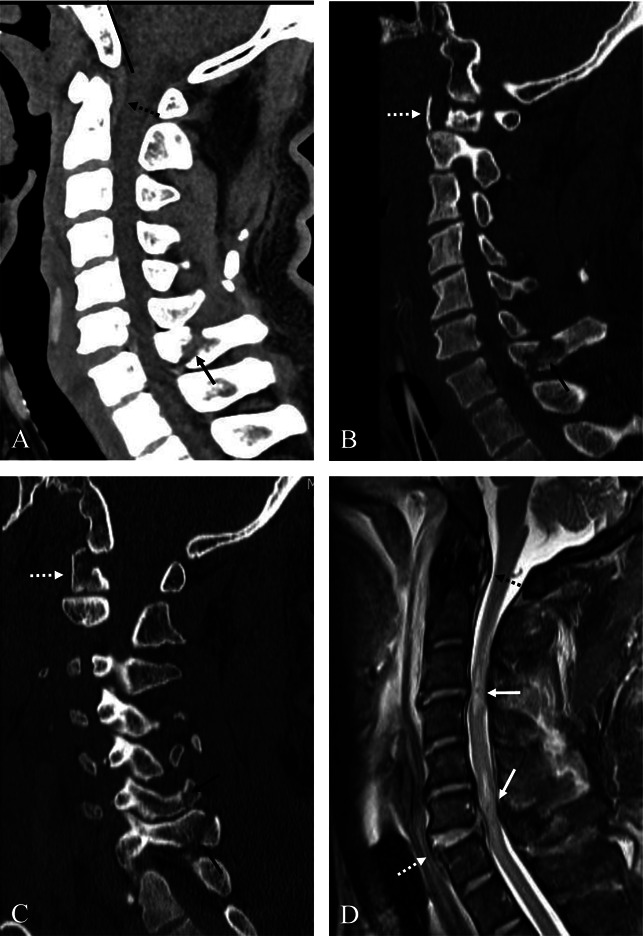
Sagittal reconstructed CT scans (**A**, **B**, and **C**) showing the direction of the Wackenheim line (dark line) behind the dens, indicating traumatic posterior atlanto-occipital dislocation (AOD), injury to the posterior ligament complex at the craniovertebral junction (CVJ) (dotted dark arrow), fracture of the C1 atlas (dotted white arrows), and fracture of the spinous process at C6 and C7 (dark arrows). Sagittal MRI (**D**) indicates traumatic intramedullary hemorrhage at C3-4 and C6-7 (white arrows), rupture of the anterior longitudinal ligament, disk, and posterior longitudinal ligament (dotted white arrows) at C3-4 and C6-7, the direction of the Wackenheim line (dark line) behind the dens, indicating traumatic posterior AOD and injury to the posterior ligament complex at the CVJ (dotted dark arrow).

**Figure 2:**
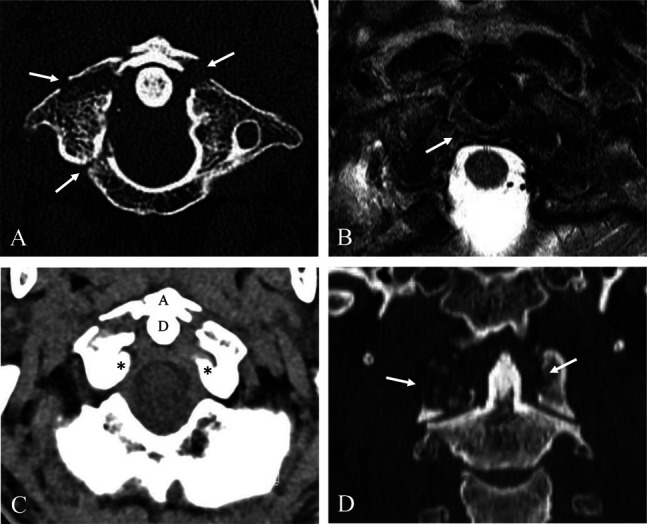
Axial CT scans (**A** and **C**) showing a three-part Jefferson fracture (white arrows) and a posteriorly displaced occiput (arrows) about the anterior arch of the atlas and the dens of the axis. Coronal reconstructed CT scan (**D**) reveals fractures of the atlas (white arrows). Axial MRI (**B**) shows the rupture of the transverse atlantal ligament at C1-2 (white arrow).

**Figure 3:**
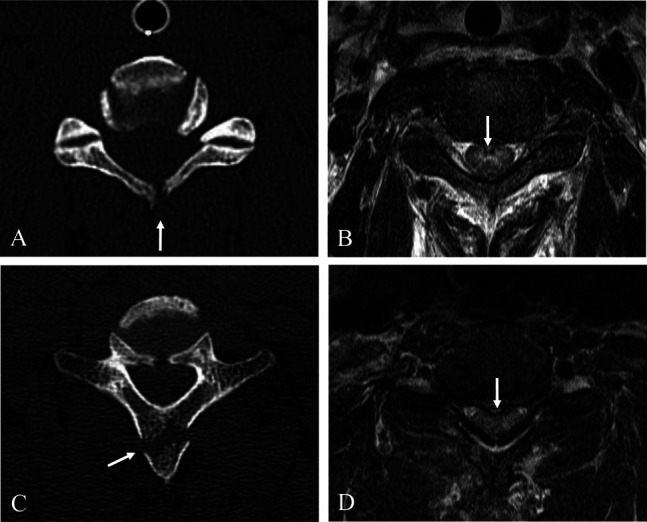
Axial CT scan (**A**) and magnetic resonance imaging (**B**) at C3-4 showing lamina fracture of C4 (white arrow) and intramedullary hemorrhage of C4 spinal cord (white arrow). Axial CT scan (**C**) and magnetic resonance imaging (**D**) at C6-7 show spinous process fracture of C7 (white arrow) and intramedullary hemorrhage of C7 spinal cord (white arrow).

A 44-year-old male patient was transferred to our emergency department from another hospital after a rear-end collision car accident. The patient was driving a passenger car on a highway, and the car had adjustable headrests, but the patient was not using his seat belt. When the patient arrived, he had already been intubated because of respiratory failure. The patient presented with quadriplegia, but intubation made it impossible to obtain a full history to identify other symptoms. CT and MRI revealed combined upper and lower cervical injuries. Sagittal reconstructed CT scans showed posterior AOD (Figure 1A), fracture of the C1 atlas (Figure 1, B and C), and fractures of the spinous processes at C6 and C7 (Figure 1, A–C). Sagittal MRI showed injury of the posterior ligament complex at the craniovertebral junction (CVJ), rupture of the anterior longitudinal ligament, disk, and posterior longitudinal ligament at C3-4 and C6-7 and traumatic intramedullary hemorrhage at C3-4 and C6-7 (Figure 1D). Axial and coronal reconstructed CT showed three-part Jefferson fracture (Figure 2, A, C, and D). Axial MRI demonstrated rupture of the transverse atlantal ligament at C1-2 (Figure 2B). Axial CT scan (Figure 3A) and MRI (Figure 3B) at C3-4 showed lamina fracture of C4 and intramedullary hemorrhage of C4 spinal cord. Axial CT scan (Figure 3C) and MRI (Figure 3D) at C6-7 showed spinous process fracture of C7 and intramedullary hemorrhage of C7 spinal cord. These CT and MRI findings suggest that a subaxial DE injury at C3-4 and C6-7 caused the spinal cord injury and resulting quadriplegia. The patient also had lung injury and pelvic bone fracture, which caused a hemodynamically unstable condition. The patient's condition was inoperable, and he died 1 day after the accident before undergoing surgical intervention.

## Discussion

Traumatic AOD results from ligamentous injury to the CVJ and is associated with a high mortality rate and notable neurologic morbidity.^1-3,6,7^ Prompt diagnosis and early surgical treatment are crucial to prevent death or further neurologic deterioration. The diagnosis of AOD has recently improved because of development of CT and MRI, but the diagnosis is sometimes overlooked if multiple traumas exist or if the vital signs are unstable.^8,9^ AOD mortality rates have decreased recently because of better prehospital and hospital management of these patients, whereas its diagnosis has improved—thanks to easier and widespread access to CT and MRI studies.^2^ Furthermore, MRI is particularly important in patients with undetermined neurological status, for example, because of associated head injuries or intubation, like our patient. Even if traumatic AOD is identified, diagnosis of associated spinal injuries may be overlooked. Therefore, high index of suspicion and careful radiologic examination are very important to avoid missing the diagnosis of associated spinal injuries in traumatic AOD, which may affect treatment, outcome, and prognosis.^10,11^

If traumatic AOD is accompanied by spinal injuries, the cause of death is typically AOD or devastating neurologic deficits. Park et al. reported a rare case of traumatic posterior AOD and Jefferson fracture with fracture dislocation of C6-7.^3^ In that case, the combined upper and lower cervical spine injuries were caused by the same injury mechanism. The most common injury mechanism that leads to traumatic posterior AOD and Jefferson fracture is axial loading and compression forces.^12,13^ The associated lower cervical spine injury was a fracture dislocation of C6-7, which was caused by a compressive flexion injury. Moreover, fracture dislocation at C6-7 was the leading cause of quadriplegia rather than traumatic posterior AOD.^3^ In this case, the traumatic posterior AOD and Jefferson fracture were the same as in the previous case reported by Park et al., but the associated lower cervical spine injury was a subaxial DE injury occurring at C3-4 and C6-7. Therefore, the most important point that distinguishes this case from the previous case is that the combined upper and lower cervical spine injuries were caused by a contrary injury mechanism in our case. It is postulated that the patient's neck was suddenly bent backward by the first injury of the rear-end collision car accident with head rest, which caused the stage 2 subaxial DE injury at C3-4 and C6-7. The following secondary neck bouncing and bending forward likely resulted in axial loading and compression forces, which seemed to cause the traumatic posterior AOD and Jefferson fracture. Both lung injury and pelvic fractures were thought to have been caused by the patient's body moving forward and hitting the steering wheel.

In conclusion, this is the first report of a case of a traumatic posterior AOD with three-part Jefferson fracture and subaxial DE injury caused by a contrary injury mechanism. Subaxial DE injury, not posterior AOD, caused fatal situation in this case. High index of suspicion and careful radiologic examination are needed to investigate the presence of associated lower cervical spine injuries caused by a contrary injury mechanism in traumatic posterior AOD, which may affect treatment, outcome, and prognosis.
